# Intrahepatic cholestasis in subclinical and overt hyperthyroidism: two case reports

**DOI:** 10.1186/1752-1947-2-116

**Published:** 2008-04-21

**Authors:** Aliye Soylu, Mustafa Gurkan Taskale, Aydin Ciltas, Mustafa Kalayci, A Baki Kumbasar

**Affiliations:** 1 Department of Gastroenterology, Dr Sadi Konuk Research Hospital, Istanbul, Turkey; 2 Department of Endocrinology and Metabolism, Dr Sadi Konuk Research Hospital, Istanbul, Turkey; 3 Department of Internal Medicine, Dr Sadi Konuk Research Hospital, Istanbul, Turkey; 4 Department of General Surgery, Dr Sadi Konuk Research Hospital, Istanbul, Turkey

## Abstract

**Introduction:**

Non-specific abnormalities in liver function tests might accompany the clinical course of hyperthyroidism. Hyperthyroidism can cause the elevation of hepatic enzymes and bilirubin. Jaundice is rare in overt hyperthyroidism, especially in subclinical hyperthyroidism. On the other hand, the use of anti-thyroid drugs has rarely been associated with toxic hepatitis and cholestatic jaundice.

**Case presentation:**

Here we present two cases of cholestasis that accompanied two distinct forms of clinical hyperthyroidism. The first patient had a clinical presentation of severe cholestasis in the absence of congestive failure related to hyperthyroidism. The second case had developed intrahepatic cholestasis in the presence of subclinical hyperthyroidism, and improved with rifampicin treatment.

**Conclusion:**

Hyperthyroidism should be a consideration in non-specific liver dysfunction.

## Introduction

Liver function test abnormalities accompanying thyroid diseases are rarely reported. Changes in liver enzyme levels can be non-specific in nature or of a cholestatic profile. In recent years, there have been rapid developments in the understanding pertaining to the pathophysiology of cholestasis secondary to thyroid disease and cholestatic injury. The mechanism underlying the hepatic damage observed in hyperthyroidism is not clear; however, since the beginning of the 20th century, different functional and histological hepatic changes have been reported in patients with thyrotoxicosis. In roughly half of hypothyroidism cases, liver function tests were found to be abnormal, whereas liver histology was normal [1,2]. In hyperthyroidism, liver function tests might reveal non-specific elevations of the transaminases, mild indirect hyperbilirubinemia (30% of cases), alkaline phosphatase (ALP) elevations (40% of patients), and there may be minor changes in liver histology. Liver enzyme elevations in thyroid disease might be paradoxically related to propylthiouracil (PTU) therapy [1-3].

We present two cases of intrahepatic cholestasis developing in thyrotoxicosis by different mechanisms, and improvement via different treatment regimens. Our aim is to remind physicians of hyperthyroidism and anti-thyroid medications in the differential diagnosis of cholestasis.

## Case presentation

### Case 1

A mass was detected on the left adrenal gland of a 47-year-old male patient who was under investigation for complaints of fatigue, loss of appetite and weight loss (20 kg) present for the previous three months. The subsequent fine-needle aspiration biopsy diagnosed a myelolipoma. During these investigations, the patient developed jaundice and pruritus, and was referred to the gastroenterology clinic for the differential diagnosis of cholestasis.

The patient had no history of chronic disease, smoking, pharmaceutical or herbal medications, infections, abuse of alcohol, blood transfusion or traveling. On initial examination, there was significant jaundice, the skin was moist and the hands were tremulous. He had tachycardia. There were no signs of thyroid ophthalmopathy. The thyroid gland was non-palpable and there were no findings of heart failure, chronic liver disease or infection. His laboratory tests showed a total bilirubin value of 15 mg/dl (normal range (N): 0.2–1.3), direct bilirubin 11 mg/dl (N: 0.1–0.5), alkaline phosphatase (ALP) 393 IU/l (N: 40–150), alanine aminotransferase (ALT) 46 IU/l (N: 0–55), aspartate aminotransferase (AST) 46 IU/l (N: 5–34), γ-glutamyl transferase (GGT) 34 IU/l (N: 9–64), lactate dehydrogenase (LDH) 399 IU/l (N: 125–243), calcium (Ca) 9.7 mg/dl (N: 8.4–10.2), phosphorus (P) 2.3–4.7 mg/dl, hemoglobin 12.5 g/dl, prothrombin time 10 seconds and α-fetoprotein (α-FP) and carcino-embryonic antigen (CEA) were within normal limits. Peripheral blood was negative for the antibody to hepatitis B surface antigen (HBsAg), immunoglobulin M antibody (IgM) to hepatitis B core antigen (anti-HBc), IgM antibody to hepatitis A virus (IgM anti-HAV), hepatitis C virus (HCV), human immunodeficiency virus (HIV), syphilis (VDRL), cytomegalovirus (CMV), Epstein-Barr virus (EBV), brucella and autoantibodies (ANA, AMA, ASMA and LKM_1_). Abdominal ultrasonography (USG) findings were normal except for leveling sludge in the gall bladder and the adrenal mass. An abdominal magnetic resonance imaging (MRI) scan revealed an adrenal mass measuring 6 × 8 × 6 cm with lobulated contours and well-defined borders; the mass was iso-intense with the retroperitoneal adipose tissue. Magnetic resonance cholangiography findings were normal. With a preliminary diagnosis of intrahepatic cholestasis, thyroid function tests were ordered and a liver biopsy was planned. Serum thyroid hormone levels were free triiodothyronine (FT_3_) 10.2 ng/dl (N: 1.8–4.2), free thyroxine (FT_4_) 5.27 ng/dl (N: 0.8–1.9), thyroid-stimulating hormone (TSH) less than 0.02 mU/ml (N: 0.4–4) and anti-thyroid peroxidase 283 (N: 0–35). In the thyroid USG, parenchymal echogenicities of both thyroid lobes were heterogeneous. Thyroid scintigraphy (3 mCi Tc 99 m pertechnetate IV) results were consistent with a diffuse hyperplasic thyroid gland that demonstrated diffuse and high-level technetium uptake. The patient was diagnosed with Graves' disease. Histopathological findings from a liver biopsy specimen were found to be consistent with acute cholestasis (cannalicular cholestasis). We considered that cholestatic jaundice had developed secondary to hyperthyroidism. PTU therapy was initiated at a daily dose of 300 mg in three divided doses. On the sixth day of PTU therapy, the patient developed pruritus together with elevations in cholestatic enzymes and bilirubin (total bilirubin: 37 mg/dl; conjugated bilirubin: 24 mg/dl), necessitating the cessation of anti-thyroid treatment. Radioactive iodine (I^131^) was administered for thyroid ablation. During follow-up, initially LDH, then total bilirubin, and lastly ALP levels gradually returned to normal values. Weekly ALP measurements during the first 45 days were 393, 409, 362, 284, 272 and 190 IU/l. Subsequent tests showed ALP levels of 162 IU/l at month 2 and 148 IU/l at month 3. Within three months, all biochemical tests were within normal limits.

### Case 2

**Figure 1:**
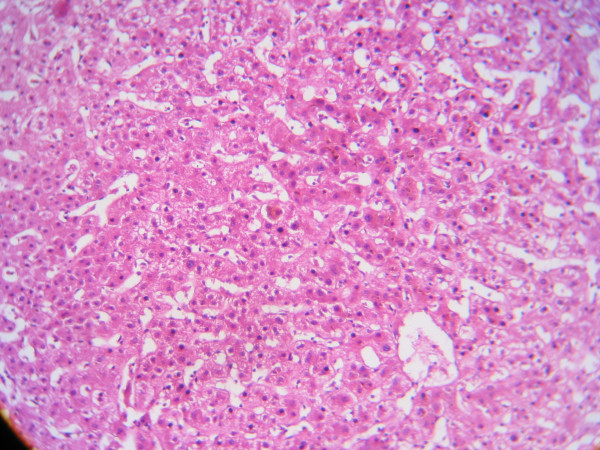
**Liver biopsy results consistent with intrahepatic cholestasis**. Hematoxylin and eosin stain, magnification ×100.

A 67-year-old female patient was admitted to hospital with complaints of pruritus and jaundice. She had no history of liver disease, and had been diagnosed with hyperthyroidism three years previously. The patient had been on anti-thyroid medications for one year, and had not experienced any hepatic problems during this period. Anti-thyroid drug therapy had been discontinued two years previously because she had reached normal thyroid status both clinically and on laboratory tests. In her laboratory results two months previously, serum thyrotropin levels were suppressed (TSH less than 0.01 uIU/ml (N: 0.4–4)), and free thyroxine was within normal limits (FT_4 _1.15 ng/dl (N: 0.9–1.7)). Our assessment was that the patient had subclinical hyperthyroidism. Six weeks prior to her admission to our clinic, she had developed pruritus; she had no history of other pharmaceutical or herbal medication use, infections, abuse of alcohol or contact with toxic substances. Physical examination findings were normal except for jaundice and the skin manifestations of pruritus. At the time of admission, her laboratory results were: AST 38 IU/l (N: 5–34), ALT 41 IU/l (N: 0–55), GGT 203 IU/l (N: 9–64), ALP 358 IU/l (N: 40–150), LDH 182 IU/l (N: 125–243), total bilirubin 24.5 mg/dl (N: 0.2–1.3), direct bilirubin 17.5 mg/dl (N: 0.1–0.5), Ca 8.8 mg/dl (N: 8.4–10.2) and P 2.3–4.7 mg/dl. Markers of viral hepatitis (HBsAg, anti-HBc IgM, anti-HAV IgM, HCV, HIV, VDRL, CMV, EBV), brucella and autoimmune hepatitis (ANA, AMA, ASMA and LKM_1_) were all negative. Endoscopic retrograde cholangiopancreatography (ERCP) and other imaging techniques did not reveal any pathology. Follow-up thyroid hormone levels were FT_3 _3.8 ng/dl, FT_4 _1.34 ng/dl (N: 1.8–4.2) and TSH 0.08 uIU/ml, correlating with subclinical hyperthyroidism. The patient's serum was negative for thyroid autoantibodies. Thyroid USG revealed a multinodular goiter pattern. The thyroid biopsy revealed nodular hyperplasia. Liver biopsy results showed degeneration and regeneration of hepatocytes, pigment accumulation in the cytoplasm of some hepatocytes, dilatation of the sinusoids, bile plugs, a slight increase in Kupffer cells, rare mononuclear inflammatory cells and regular appearance of bile ductules. This was consistent with intrahepatic cholestasis (Figure 1).

The symptoms of jaundice and pruritus did not respond to an 18-day course of treatment with corticosteroids, ursodeoxycholic acid or other symptomatic treatment. We added rifampicin at a dose of 600 mg/day. On the third day of rifampicin therapy, cholestasis parameters started to regress and were significantly improved by day 10. Weekly measurements during rifampicin use revealed ALP levels of 358, 349, 241 and 166 IU/l, and at month 2 ALP levels had declined to 103 IU/l. In the second month of therapy, clinical and cholestasis-related laboratory findings were within normal limits. The patient was not placed under specific treatment due to the subclinical course of the disease, but frequent follow-up of thyroid function tests were recommended.

## Discussion

The first patient had presented with cholestasis secondary to autoimmune hyperthyroidism, and his jaundice was aggravated by anti-thyroid medications. He was treated with radioactive iodine (I^131^), and the jaundice improved when euthyroidism was achieved after three months. In the second patient, cholestatic jaundice was caused by subclinical hyperthyroidism and treated with rifampicin. However, rifampicin use can also cause intrahepatic cholestasis [3]. The issues raised by these cases include the etiology of liver enzyme abnormalities, the elevation of serum bilirubin levels in hyperthyroidism and the clinical management of these liver abnormalities.

The liver is the primary organ of thyroid hormone metabolism. This partly explains the complexity of the influences of increased thyroid hormone levels on liver function tests. Individual differences are attributed to liver enzyme levels [4].

The co-occurrence of hyperthyroidism and abnormal liver function tests is rare and the mechanism underlying hepatic dysfunction is not well known. In etiology, enzyme induction and the role of venous congestion due to heart failure are stipulated. Hypoxia is blamed as another mechanism and it is anticipated that increased oxygen utilization cannot be totally compensated for by hepatic blood flow. In patients with high levels of T_3 _and T_4_, relatively severe hypoxemia develops and the pericentral parts of hepatic acini become prone to damage. The mechanism underlying hyperbilirubinemia is not well known either and there are no available data supporting the direct toxic effects of thyroid hormones on the liver [5].

Cholestasis secondary to medication and chemical agents is on the rise for liver diseases [1]. With such feedback, there has been a rapid advance in information pertaining to the pathophysiology of cholestasis and the mechanism of cholestatic damage. Injury to hepatocytes and disturbances in the secretion and flow of bile has been blamed in cholestasis secondary to medications. Anti-thyroid medications are listed among pharmacological agents that cause cholestasis [6]. In our first case, parameters of cholestasis had amplified further with the initiation of PTU therapy and ceased to intensify upon discontinuation. This pattern was associated with the cholestatic effect of PTU.

Liver pathologies ranging from mild to fulminant liver failure with a fatal course have been reported with PTU use [7]. The liver biopsies of three asymptomatic patients on PTU therapy with ALT elevations were reported as focal necrosis or ill-defined granuloma composed of foamy histiocytes with ceroid pigment and mild fatty metamorphosis [8]. In both of our patients, signs of intrahepatic cholestasis existed in their liver biopsies, and pathologies with an inflammatory process were excluded.

Liver biopsies of five patients [8] with hyperthyroidism revealed non-specific changes such as mild to moderate intrahepatic cholestasis, lobular inflammation of eosinophilic origin and Kuppfer cell hyperplasia. There was no correlation between the severity of histological damage and thyroid function tests.

Jaundice can be diagnosed during the clinical course of thyrotoxicosis while mild increases are observed in ALT, AST and bilirubin levels. In a study by Thompson et al [9], abnormal function tests and, in particular, elevations of bilirubin were reported in hyperthyroidism. Despite the rarity of case presentations of intrahepatic cholestasis caused by subclinical hyperthyroidism, the condition might present clinically with findings of severe cholestasis. One report presented a case of severe intrahepatic cholestasis due to toxic multinodular goiter, with normal FT_3 _and FT_4 _levels, without congestive heart failure, and with subclinical hyperthyroidism [10]. After the exclusion of other possible causes of intrahepatic cholestasis, we concluded that our second case was related to subclinical hyperthyroidism in the absence of congestive heart failure.

Although adverse reactions of anti-thyroid medications are not reported frequently, methimazole (MMI) – and PTU-related cases have been observed at equal frequencies [11]. PTU-related hepatocellular and MMI-related cholestatic hepatitis cases have been reported [8,12]. The adverse effects of MMI are related to the dose [11] or of immunological origin. Side-effects generally appear during the first three months of treatment, but they may also arise much later in the course of treatment [7].

In Woeber's review [12], 30 previously published cases of patients who had developed hepatotoxicity following treatment with MMI and carbimazole were analyzed; 19 had cholestatic-form clinical presentations. Clinical improvement is slow, yet the condition is totally reversible. An analysis of the cases reported by Woeber demonstrated that advanced age and high-dose anti-thyroid medications were risk factors for cholestatic damage [12]. In one study, asymptomatic elevations of GGT and ALP were observed in one-third of patients receiving PTU treatment, and 75.8% were identified as having at least one biochemical abnormality. AST was elevated in 27.4% of cases, ALT in 36.8%, ALP in 64.2%, GGT in 16.8% and bilirubin in 5.3% [13].

In our first case, cholestatic jaundice caused by overt hyperthyroidism was aggravated as an adverse effect of PTU. We administered radioactive iodine (I^131^) for thyroid ablation and the patient improved. Thyrotoxic cholestasis might develop as a result of diverse mechanisms and is corrected with the establishment of euthyroidism. Patients should be warned about the side-effects of medications when anti-thyroid therapy is to be administered. They should be followed up for hepatotoxicity and advised to stop treatment if symptoms develop [7]. Treatment can be maintained in patients who do not have symptoms or hyperbilirubinemia. Anti-thyroid medication-related pathologies are frequently benign and transient, and side-effects are reversible in nearly all patients [7,8].

If intrahepatic cholestasis refractory to other treatments develops in cases of subclinical hyperthyroidism, patients may be placed under observation without anti-thyroid treatment. Rifampicin can be considered as an alternative treatment choice. It is a pregnane X receptor (PXR) agonist, decreasing the synthesis of bile acids by activating PXR. PXR is a nuclear receptor predominantly expressed in the liver and bowel; it coordinates the response of the liver to environmental toxins. Activated PXR is anti-cholestatic and selective PXR agonists can be used in the treatment of advanced cholestasis. The inhibition of bile acids with rifampicin can be a protective mechanism for medication- and bile acid-related cholestasis [14,15]. Owing to such effects, rifampicin is recommended in cholestasis-related pruritus at a dose of 300 mg twice daily [3]. The second case, whose pruritus and jaundice were unresponsive to any symptomatic therapy, showed a rapid response to treatment with rifampicin.

## Conclusion

In non-specific abnormalities of liver function tests, hyperthyroidism should be considered and the patient's thyroid function should be assessed.

## Competing interests

The authors declare that they have no competing interests.

## Authors' contributions

AS provided patient care, performed liver biopsies and prepared the manuscript. MGT was involved in endocrinological care. AC carried out the literature review. MK conducted surgical consultations. ABK was involved in patient care and treatment maintenance. All authors read and approved the final manuscript.

## Consent

Written informed consent was obtained from the patients for publication of this case report and accompanying images. A copy of the written consent is available for review by the Editor-in-Chief of this journal.
